# Identification of the Amino Acids 300–600 of IRS-2 as 14-3-3 Binding Region with the Importance of IGF-1/Insulin-Regulated Phosphorylation of Ser-573

**DOI:** 10.1371/journal.pone.0043296

**Published:** 2012-08-17

**Authors:** Sabine S. Neukamm, Rachel Toth, Nick Morrice, David G. Campbell, Carol MacKintosh, Rainer Lehmann, Hans-Ulrich Haering, Erwin D. Schleicher, Cora Weigert

**Affiliations:** 1 Division of Clinical Chemistry and Pathobiochemistry, Department of Internal Medicine IV, University Hospital Tuebingen, Tuebingen, Germany; 2 Institute for Diabetes Research and Metabolic Diseases of the Helmholtz Centre Munich at the University of Tuebingen (Paul Langerhans Institute Tuebingen), Member of the German Diabetes Centre (DZD e.V.), Tuebingen, Germany; 3 Division of Signal Transduction and Therapy, College of Life Sciences, University of Dundee, Dundee, Scotland, United Kingdom; 4 Beatson Institute for Cancer Research, University of Glasgow, Glasgow, Scotland, United Kingdom; 5 MRC Protein Phosphorylation Unit, College of Life Sciences, University of Dundee, Dundee, Scotland, United Kingdom; 6 Division of Endocrinology, Diabetology, Vascular Medicine, Nephrology and Clinical Chemistry, Department of Internal Medicine IV, University Hospital Tuebingen, Tuebingen, Germany; Virgen Macarena University Hospital, School of Medicine, Spain

## Abstract

Phosphorylation of insulin receptor substrate (IRS)-2 on tyrosine residues is a key event in IGF-1/insulin signaling and leads to activation of the PI 3-kinase and the Ras/MAPK pathway. Furthermore, phosphorylated serine/threonine residues on IRS-2 can induce 14-3-3 binding. In this study we searched IRS-2 for novel phosphorylation sites and investigated the interaction between IRS-2 and 14-3-3. Mass spectrometry identified a total of 24 serine/threonine residues on IRS-2 with 12 sites unique for IRS-2 while the other residues are conserved in IRS-1 and IRS-2. IGF-1 stimulation led to increased binding of 14-3-3 to IRS-2 in transfected HEK293 cells and this binding was prevented by inhibition of the PI 3-kinase pathway and an Akt/PKB inhibitor. Insulin-stimulated interaction between endogenous IRS-2 and 14-3-3 was observed in rat hepatoma cells and in mice liver after an acute insulin stimulus and refeeding. Using different IRS-2 fragments enabled localization of the IGF-1-dependent 14-3-3 binding region spanning amino acids 300–600. The 24 identified residues on IRS-2 included several 14-3-3 binding candidates in the region 300–600. Single alanine mutants of these candidates led to the identification of serine 573 as 14-3-3 binding site. A phospho-site specific antibody was generated to further characterize serine 573. IGF-1-dependent phosphorylation of serine 573 was reduced by inhibition of PI 3-kinase and Akt/PKB. A negative role of this phosphorylation site was implicated by the alanine mutant of serine 573 which led to enhanced phosphorylation of Akt/PKB in an IGF-1 time course experiment. To conclude, our data suggest a physiologically relevant role for IGF-1/insulin-dependent 14-3-3 binding to IRS-2 involving serine 573.

## Introduction

Insulin and insulin-like growth factor (IGF)-1 mediate their metabolic and mitogenic effects on target tissues through activation of the IGF-1/insulin receptor. The activated receptors function as tyrosine kinases that phosphorylate proteins including the insulin receptor substrate proteins (IRS)-1 and -2 [1], [2]. This leads to activation of the PI 3-kinase-Akt/PKB pathway and promotes glucose and lipid storage, protein synthesis and cell survival. The mitogenic effects are mediated by the activation of the Ras/MAPK pathway, leading to differentiation and proliferation. The signal diversification is modulated by the IRS proteins and their phosphorylation status. After being phosphorylated on tyrosine residues the IRS proteins serve as docking molecules for src homology 2 (SH2) domain containing intracellular signaling proteins, e.g. p85 regulatory subunit of PI 3-kinase [3], [4]. IRS-1 and IRS-2 proteins also are substrates for a variety of serine/threonine kinases [5]–[12]. Given the essential and complementary functions of IRS-1 and IRS-2 in transducing and terminating the IGF-1/insulin signal it is evident that dysregulation of serine/threonine phosphorylation can have important consequences for cellular metabolism and cell survival. Alignment of IRS-1 and -2 protein sequences shows a high sequence identity and conservation of some but not all serine and threonine residues. The site-specific regulation and function of the phosphorylated serine/threonine residues in IRS proteins make it necessary to identify and characterize the individual phospho-sites in the individual proteins. Notably, hyperphosphorylation of serine/threonine residues in IRS-1 is a key event in the development of insulin resistance [1], [13], [14], while the phosphorylation of certain residues can enhance insulin signaling [8], [15], [16]. Less is known about individual phospho-sites in IRS-2 and the (patho)physiological consequences of serine/threonine phosphorylation of IRS-2 [17].

14-3-3 proteins are highly conserved and expressed in a wide range of eukaryotes. The high level of functional conservation of the 14-3-3 proteins is indicated by the finding that the isoforms from yeast, plant and mammals are functionally interchangeable [18]. In humans seven genes have been identified to encode for seven 14-3-3 isoforms. They form homo- or heterodimers with the exception of 14-3-3σ, which preferentially forms homodimers [19]. Studies with dimerization-deficient 14-3-3 mutants revealed a significant role for dimerization, that seems to be necessary for phosphorylation-dependent interaction with target proteins [18]. Crystal structures show that each 14-3-3 protein resembles a curved structure with a central groove and within this central groove the residues are strictly conserved [20]. The central groove enables 14-3-3 proteins to bind to a phosphorylated serine or threonine residue within specific motifs in target proteins, termed mode I (RSXpS/pTXP, X denotes any amino acid) and mode II (RX(F/Y)XpS/pTXP) [21], [22]. The phosphorylation of serine and threonine sites in 14-3-3 binding partners is an important prerequisite for the interaction although the aforementioned motifs are not completely mandatory and in rare cases phosphorylation is not required [23]–[25]. There are studies reporting that interaction between 14-3-3 and the target protein is based upon phosphorylation of a single serine/threonine residue [26]–[28], but there are also studies that found two simultaneously phosphorylated serine/threonine residues to mediate interaction with 14-3-3 proteins [29]–[31]. 14-3-3 proteins can act in several ways: conformational change of the target protein [32], blocking of sequence specific or structural features [33], scaffolding of protein clusters to connect signaling pathways [34] and intracellular trafficking [35]. Of note, the possibility that more than one mode of action is used simultaneously is likely.

The abundance of 14-3-3 proteins and their broad range of interaction partners explain why they are key regulators of various intracellular processes, such as neuronal development, cell cycle control, cell growth control, apoptosis and control of gene transcription. Recently, IRS-2 was identified by a differential 14-3-3 affinity capture approach as novel 14-3-3 binding protein regulated by IGF-1/insulin signaling [36]. Interaction between IRS-1 and 14-3-3 has been described before, but since IRS-1 and -2 have similar and distinct properties the characterization of interaction between 14-3-3 and IRS-2 has to be investigated. It is likely that the binding of 14-3-3 to IRS-2 plays an important role in IGF-1/insulin signal transduction and that IGF-1/insulin-induced phosphorylation of certain serine/threonine residues in IRS-2 enables the 14-3-3 interaction. The aim of the present study was to identify IGF-1/insulin-dependent serine/threonine phosphorylation sites of IRS-2 with a special focus to elucidate and characterize the 14-3-3 binding sites on IRS-2.

## Results

### Identification of Phosphorylated Serine/threonine Residues on IRS-2 by Mass Spectrometry

**Figure 1:**
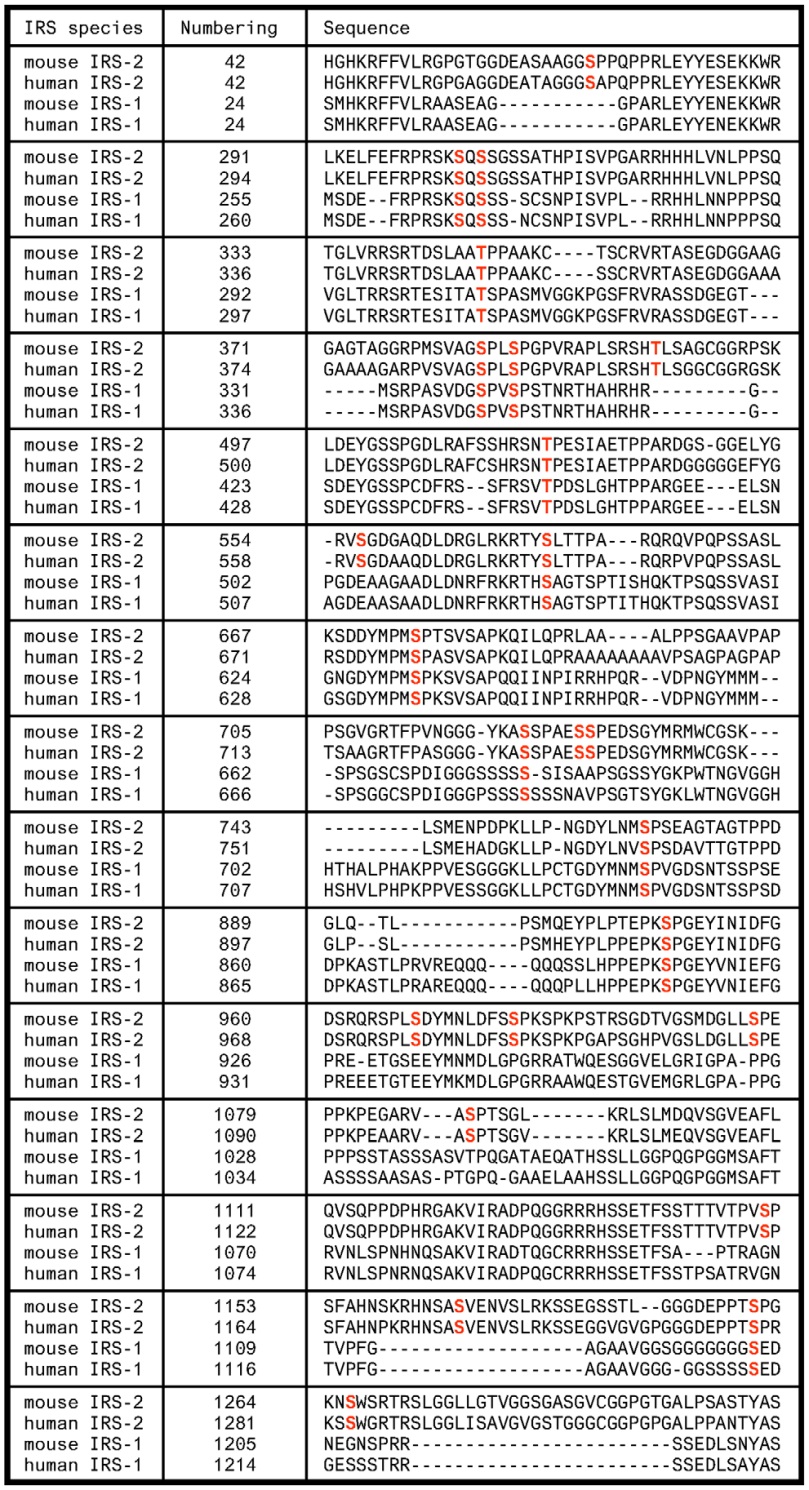
Sequence alignments of IRS-1 and -2 species. Protein sequences from mouse IRS-2 (NP_001074681.1), human IRS-2 (NP_003740.2), mouse IRS-1 (NP_034700.2) and human IRS-1 (NP_005535.1) were aligned. The sequences were searched for homologue residues of mouse IRS-2 that were identified by mass spectrometry and marked in bold red. Shown are only parts of the sequences that contain an identified phosphorylated serine/threonine residue. The term “numbering” indicates the amino acid position of the first amino acid in every line.

To identify the serine/threonine residues of IRS-2 that are involved in the IGF-1/insulin signaling cascade GFP-IRS2 encoding mouse IRS-2 was expressed transiently in HEK293 cells and cells were either left unstimulated or stimulated with IGF-1 for 30 min. Following purification, SDS-PAGE and tryptic digestion of IRS-2 protein bands liquid chromatography mass spectrometry (LC-MS) was applied to identify phosphorylated peptides. Mass spectrometric fragmentation pattern were used to localize the phosphorylation sites. The sequence coverage was 92% and a total of 24 phosphorylated serine/threonine residues were identified. According to IRS-2 mouse numbering phosphorylations were detected at the residues Ser-66, Ser-303, Ser-305, Ser-347, Ser-385, Ser-388, Thr-401, Thr-517, Ser-556, Ser-573, Ser-675, Ser-722, Ser-727, Ser-728, Ser-762, Ser-907, Ser-968, Ser-977, Ser-999, Ser-1089, Ser-1151, Ser-1165, Ser-1190 and Ser-1266 (see Table S1 and S2 for the identified phosphopeptides). In Figure 1 the sections of the IRS-2 mouse sequence are shown that contain the 24 phosphorylated residues (labeled in bold red). Corresponding residues found in human IRS-2 and mouse/human IRS-1 are also shown. Of note, Ser-305 and Ser-1266 were the only sites that were unique to the IGF-1 treated sample. However, our mass spectrometric approach was suitable to identify phosphorylation sites, but did not allow evaluation of changes in the phosphorylation levels due to IGF-1 treatment. Interestingly, some residues are unique for IRS-2 (Ser-66, Thr-401, Ser-556, Ser-727, Ser-728, Ser-968, Ser-977, Ser-999, Ser-1089, Ser-1151, Ser-1190 and Ser-1266), whereas some are conserved in IRS-1 and -2 (Ser-303, Ser-305, Thr-347, Ser-385, Ser-388, Thr-517, Ser-573, Ser-675, Ser-722, Ser-762, Ser-907 and Ser-1165).

### Co-immunoprecipitation and Overlay Assay Indicate Interaction of 14-3-3 and IRS-2 upon IGF-1 and Insulin Stimulation

**Figure 2:**
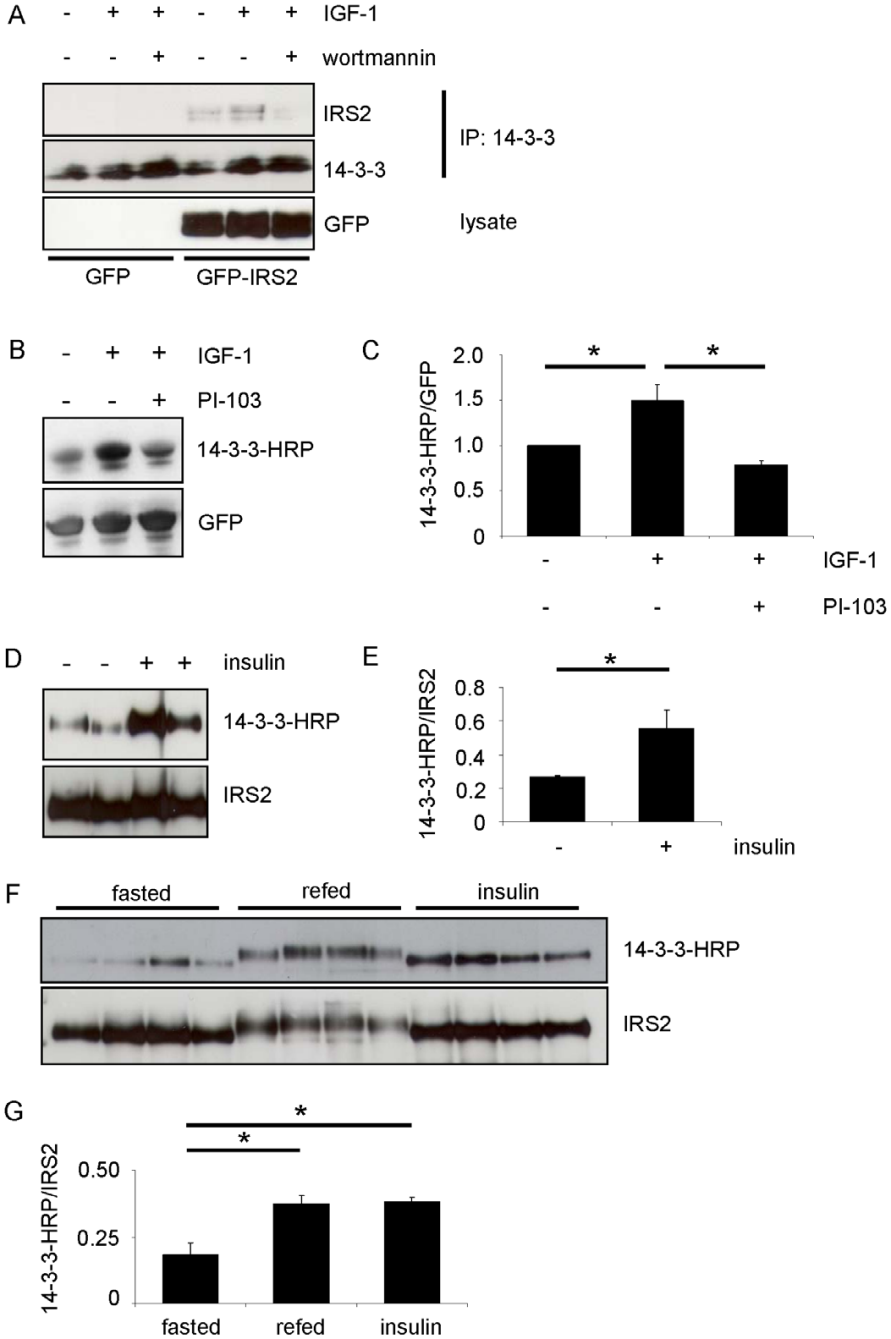
Co-immunoprecipitation and overlay assays indicate interaction of 14-3-3 and IRS-2 upon IGF-1/insulin stimulation. *A.* HEK293 cells were transiently transfected with GFP or GFP-IRS2 and after serum starvation cells were incubated with 50 ng/ml IGF-1 for 30 min or after preincubation with 100 nM wortmannin for 30 min. 400 µg total protein was used for immunoprecipitation with 14-3-3 antibody (C-17) and samples were separated on 5–15% gradient gel. Upper membrane was incubated with IRS-2 antibody, lower membrane with 14-3-3 antibody (K-19). *B.* HEK293 cells were transfected with GFP-IRS2 and stimulation was carried out after starvation for serum overnight with 50 ng/ml IGF-1 for 30 min or subsequently after preincubation with 1 µM PI-103 for 30 min. 250 µg of total protein was pulled down using GFP-Trap®. SDS-PAGE followed transfer onto nitrocellulose membranes. Overlay assay followed stripping of the membrane and reprobing with GFP antibody as loading control. *C.* Extent of interaction was quantified by scanning densitometry of blots and normalization for GFP-IRS2 serum starved condition (mean ± SEM; n = 4; *p<0.05 serum starved vs. IGF-1 or IGF-1 vs. PI-103/IGF-1). *D*. Male C57Bl/6 mice were fasted overnight and injected intravenously with 2 IU (international units) insulin. After 10 min liver was taken and 500 µg of total protein was immunoprecipitated with IRS-2 antibody. After performing overlay assay membrane was stripped and reprobed with IRS-2 antibody as loading control. Two mice of each group are shown. *E*. Densitometric analyses of 14-3-3 interaction with IRS-2. Overlay signal was normalized for total IRS-2 protein content (mean ± SEM; n = 4; *p<0.05 fasted vs. insulin). *F*. Male C57Bl/6 mice were fasted overnight, refed for 4 hours or injected intraperitoneally with insulin for 30 min. Procedure as in *G*. Four mice of each group are shown. *G*. 14-3-3 interaction with IRS-2 was quantified by scanning densitometry of immunoblots and normalization for IRS-2 protein (mean ± SEM; n = 4; *p<0.05 fasted vs. refed and insulin stimulation).

IGF-1 stimulation leads to interaction of IRS-2 and 14-3-3 as has been shown by Ogihara et al. and Dubois et al. [36], [37]. It is known that the majority of interactions between 14-3-3 proteins and their partners are dependent on phosphorylation of serine/threonine residues within discrete motifs. In a first experiment the interaction between IRS-2 and 14-3-3 was verified in HEK293 cells transiently expressing GFP-IRS2. Immunoprecipitation of endogenous 14-3-3 also pulled down IRS-2 as indicated by western blot (Figure 2A). IGF-1 stimulation increased the amount of IRS-2 that was pulled down, whereas incubation with the covalent PI 3-kinase inhibitor wortmannin decreased binding between 14-3-3 and IRS-2. Direct protein-protein interaction was visualized by overlay assay and showed that upon stimulation with IGF-1 for 30 min the 14-3-3 binding to IRS-2 was significantly increased, whereas it was significantly decreased by treating cells 30 min prior IGF-1 stimulation with another PI 3-kinase inhibitor, namely PI-103, which targets the catalytic subunit p110 of PI 3-kinase (Fig. 2B, C). These data suggested that the activation of the PI 3-kinase pathway induced phosphorylation on the IRS-2 molecule on serine or threonine residues within a 14-3-3 binding motif. To evaluate the physiological importance of 14-3-3 binding to IRS-2 we tested if the overlay assay could show 14-3-3 binding to IRS-2 in mouse liver after an acute insulin stimulus. Mice were fasted overnight, injected intravenously with insulin and after 10 min liver was taken and IRS-2 was immunoprecipitated. The overlay assay in Figure 2D and the densitometric analysis thereof (Fig. 2E) showed a significant increase in the ability of 14-3-3 to bind to IRS-2. Another set of mice was fasted overnight and either refed for 4 hours or injected intraperitoneally with insulin for 30 min before liver was taken and IRS-2 was immunoprecipitated. Again, 14-3-3 showed increased binding to IRS-2 after the acute insulin stimulus in the overlay assay, but also the refeeding condition led to an increased binding of 14-3-3 to IRS-2 when the overlay signal was normalized against total protein content (Fig. 2F, G). Of note, the differing molecular weight of the IRS-2 protein in the refed state indicates that 30 min of insulin stimulation did not lead to such extensive posttranslational modifications on the IRS-2 protein as the physiological stimulus of refeeding over 4 hours did. In conclusion, refeeding led to increased interaction between IRS-2 and 14-3-3, thus implicating a physiological importance of this interaction.

### Localization of the 14-3-3 Binding Region on IRS-2

**Figure 3:**
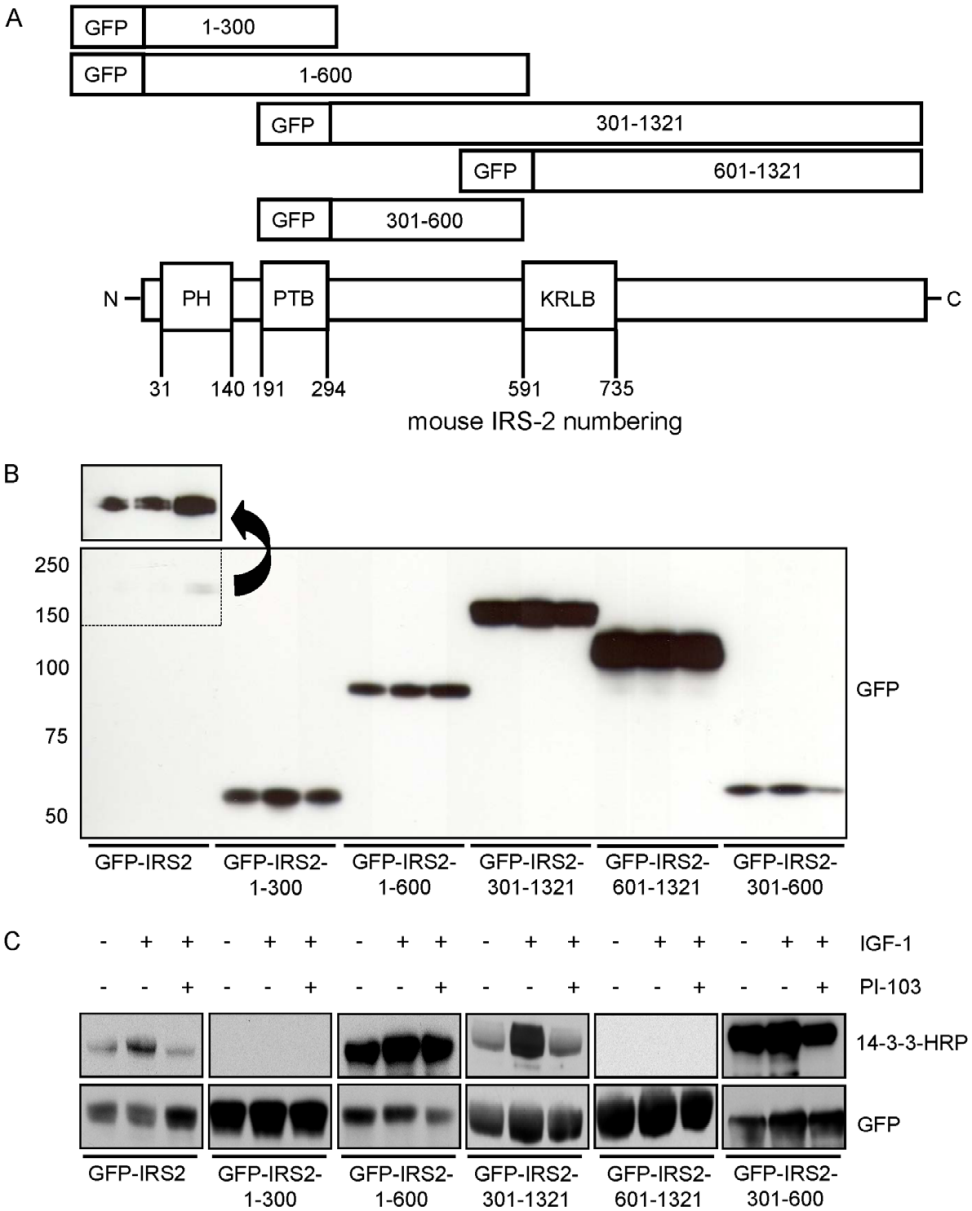
The area spanning amino acids 301–600 on IRS-2 is the 14-3-3 binding region. *A.* Schematic illustration of truncated IRS-2 constructs to identify the 14-3-3 binding region. *B.* 20 µg of protein was separated on a 5–15% gradient gel and membrane was probed with GFP antibody to check expression and molecular weight of truncated IRS-2 versions. The arrow indicates a longer exposure time. *C.* HEK293 cells were transfected with either GFP-IRS2 or truncated versions of IRS-2 (GFP-IRS2-1-300, GFP-IRS2-1-600, GFP-IRS2-301-1321, GFP-IRS2-601-1321, GFP-IRS2-301-600), starved for serum overnight and stimulated for 30 min with 50 ng/ml IGF-1 or subsequently after preincubation with 1 µM PI-103 for 30 min. With GFP-Trap® 250 µg protein was pulled down and samples were subjected to overlay assay. For loading and expression control membranes were stripped and reprobed for GFP.

Next, we aimed to localize the binding site that mediated 14-3-3 binding to IRS-2. By comparing the 14-3-3 binding motifs RSXpS/pTXP and RX(F/Y)XpS/pTXP with the sequences surrounding the identified phosphorylated residues it became apparent that potential 14-3-3 binding motifs frequently appeared in an amino acid area between the PTB (phosphotyrosine binding) and the KRLB (kinase regulatory loop binding) domain on IRS-2 stretching from amino acid position 300–600. In Table 1 the 5 residues with surrounding amino acid sequence that corresponded to a 14-3-3 binding motif are shown. To prove that region 300–600 was important for 14-3-3 binding and to exclude possible other regions IRS-2 fragments spanning different sections of the protein were generated. In Figure 3A a schematic illustration of the generated fragments is displayed. Two fragments from position 1–300 and 1–600 encompassed the N-terminal part of the IRS-2 molecule including the PH (pleckstrin homology) and the PTB domain. The C-terminal part was also divided into two fragments spanning amino acids 301–1321 and 601–1321, both containing the KRLB domain. The 5^th^ construct comprised amino acids 301–600. Constructs were GFP tagged at the N-terminus and expressed transiently in HEK293 cells to check for residual binding of 14-3-3 by overlay assay. While equal amounts of DNA were used to transfect the constructs, the protein expression levels differed between IRS-2 wild type and truncated IRS-2 forms (Fig. 3B). The overlay assay showed regulated binding of 14-3-3 to wild type IRS-2, but no interaction between 14-3-3 and the constructs GFP-IRS2-1-300 and GFP-IRS2-601-1321 (Fig. 3C). A strong and almost unregulated interaction was observed with the construct GFP-IRS2-1-600. On the contrary, the construct GFP-IRS2-301-1321 showed regulated 14-3-3 binding upon IGF-1 stimulation and reduced binding by PI-103 treatment. These data showed the importance of the region 301–600 for 14-3-3 binding. To test if this region alone was capable of binding to 14-3-3 the fragment 301–600 was tested (Fig. 3C). This fragment also showed almost unregulated binding to 14-3-3 similar to GFP-IRS2-1-600.

**Table 1:** With mass spectrometry identified residues with surrounding amino acids that correspond to a 14-3-3 binding motif.

residue	peptide
303	RSKRSK**pS**QSSGSS
401	PLSRSH**pT**LSAGCG
517	SSHRSN**pT**PESIAE
556	RPYRRV**pS**GDGAQD
573	LRKRTY**pS**LTTPAR

Shown are the identified residues indicated in bold with pS or pT and the surrounding 6 amino acids before and after the phosphorylated residue.

### Identification of Serine 573 as IGF-1-dependent 14-3-3 Interaction Site on IRS-2

**Figure 4:**
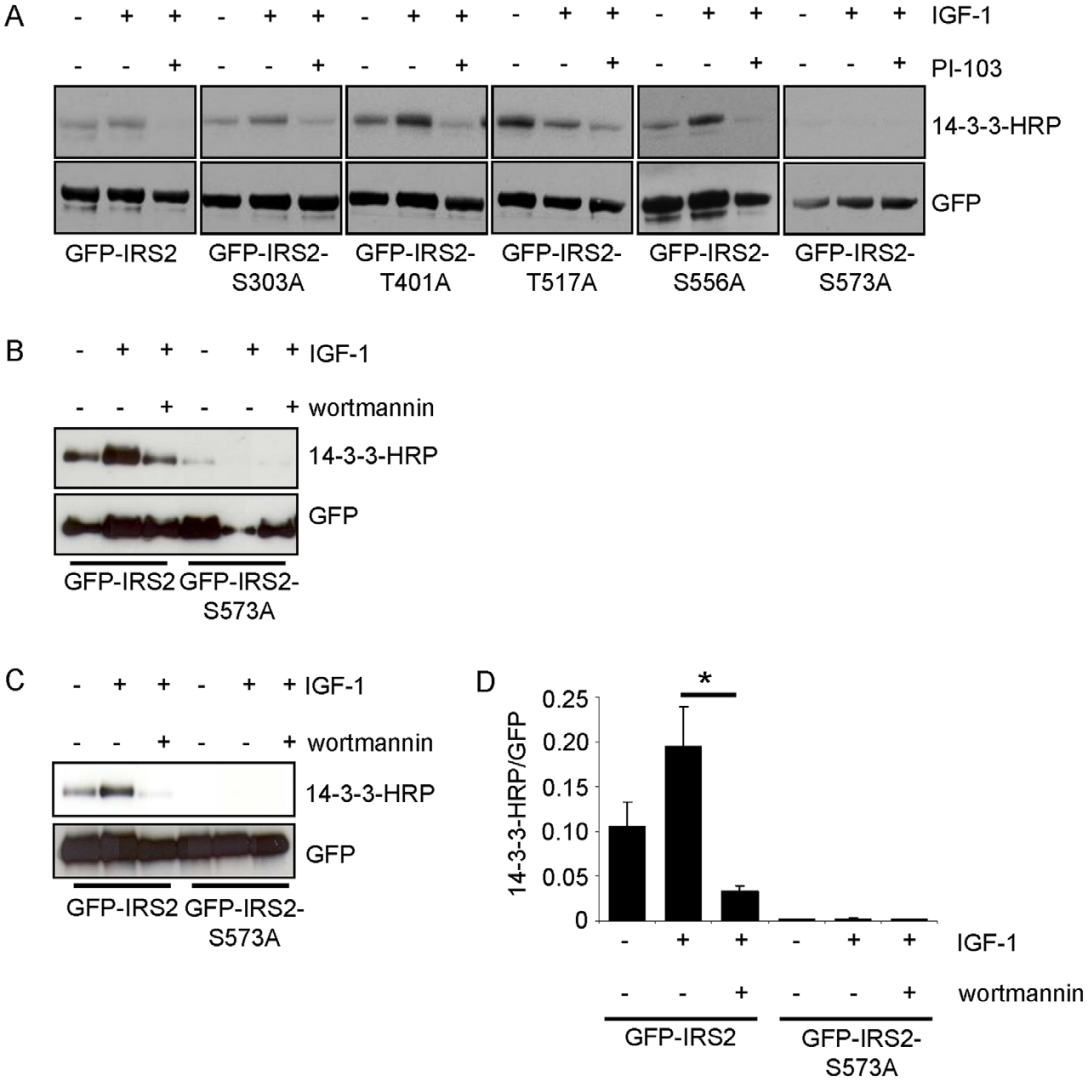
Ser-573 of IRS-2 is an IGF-1-dependent 14-3-3 binding site. *A.* HEK293 cells were transiently transfected with GFP-IRS2, GFP-IRS2-S303A, GFP-IRS2-T401A, GFP-IRS-2-T517A, GFP-IRS2-S556A or GFP-IRS2-S573A and stimulated with 50 ng/ml IGF-1 for 30 min or after preincubation with 1 µM PI-103 30 min prior IGF-1 stimulation. 250 µg of total protein were used for GFP pulldown and overlay assay was performed. Stripping followed reprobing of the membrane with GFP antibody as loading and expression control. *B.* Transiently with GFP-IRS2 or GFP-IRS2-S573A transfected HEK293 cells were stimulated with 50 ng/ml IGF-1 alone for 30 min or after preincubation with 100 nM wortmannin for 30 min. 100 µg of total protein was used for GFP pulldown, separated on 5–15% SDS gels and overlay assay was performed. Corresponding GFP reblot as expression and loading control is shown *C*. Flp-In HEK293 cells stably expressing GFP-IRS2 or GFP-IRS2-S573A were stimulated as in *A* and cell lysis was followed by pull down of 400 µg total protein. Samples were divided into two equal volumes and separated on SDS-PAGE. One membrane was used for overlay assay, the other one was directly incubated with GFP antibody. *D*. Interaction of 14-3-3 with IRS-2 from *B* was quantified scanning densitometry of Western blots (mean ± SEM; n = 3; *p<0.05 IRS-2 wild type IGF-1 vs. IRS-2 wild type wortmannin/IGF-1).

Having localized the 14-3-3 binding region to amino acids 301–600 we focused on the phosphorylated serine/threonine residues that were identified by mass spectrometry and corresponded to a 14-3-3 binding motif as shown in Table 1. After single mutation of each serine/threonine to alanine residual 14-3-3 binding upon IGF-1 stimulation was tested in transiently transfected HEK293 cells by overlay assay (Fig. 4A). The mutation of serine 573 to alanine blocked the basal and IGF-1-dependent 14-3-3 interaction completely, while the other mutants showed similar interaction with 14-3-3 when compared with IRS-2 wild type (Fig. 4A, B). Flp-In HEK293 cells were used for stable transfection with GFP-IRS2 or GFP-IRS2-S573A and showed similar results (Fig. 4C, D). Notably, no difference in the ability of the two PI 3-kinase inhibitors used (PI-103 in Fig. 4A, wortmannin in Fig. 4B, C) to decrease IGF-1-mediated 14-3-3 binding to IRS-2 was detected.

### Sequence Alignments of Different IRS-2 Species and Generation of a Phosphospecific Antibody Against Ser-573

**Figure 5:**
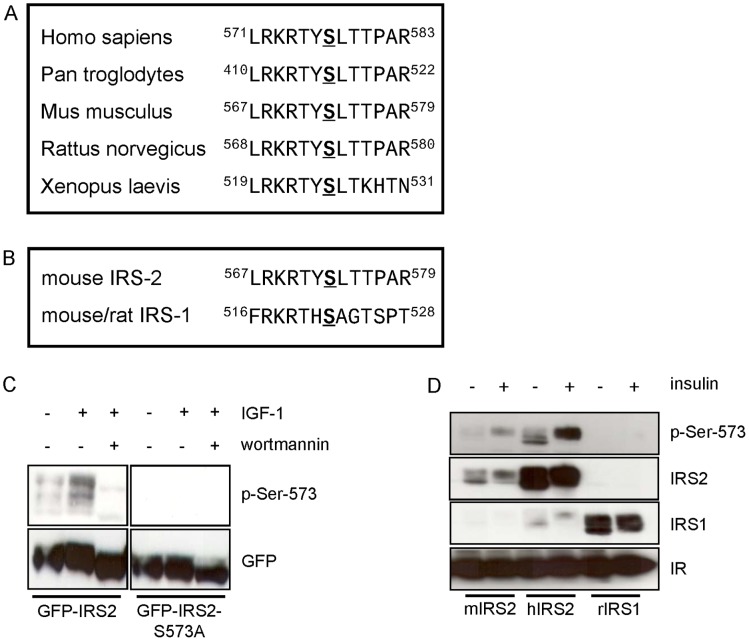
Sequence alignments and characterization of a polyclonal antibody raised in sheep against position Ser-573 on IRS-2. *A.* Sequence alignment of the amino acids adjacent to the 14-3-3 binding site Ser-573 of IRS-2 in different species. *B*. Shown is the amino acid sequence surrounding Ser-573 in mouse IRS-2 and the sequence surrounding the homologue position Ser-522 in mouse/rat IRS-1. *C*. Flp-In HEK293 cells stably expressing GFP-IRS2 or GFP-IRS2-S573A were starved for serum followed by stimulation for 30 min with 50 ng/ml IGF-1 or after preincubation with 100 nM wortmannin for 30 min. 200 µg of lysate was separated on a 7.5% SDS gel, membrane was probed with p-Ser-573 antibody. Expression was checked by stripping the membrane and reprobing with GFP antibody. *D*. HEK293 cells were co-transfected transiently with mouse IRS-2/IR (insulin receptor), human IRS-2/IR and rat IRS-1/IR and stimulated with 10 nM insulin for 30 min. Membranes were incubated with p-Ser-573 antibody and reprobed with the corresponding protein antibodies. IR expression was also checked.

Aligning of IRS-2 protein sequences from mammalian and amphibian species showed that serine 573 and the flanking motif is highly conserved (Fig. 5A). Sequence alignment of mouse/rat IRS-1 and IRS-2 showed serine 522 of IRS-1 is homologue to serine 573 of IRS-2 (Fig. 5B). To proceed with the characterization of the 14-3-3 binding site serine 573 a phospho-site specific antibody was generated. To test the specificity of the antibody Flp-In HEK293 cells stably expressing GFP-IRS2 or GFP-IRS2-S573A were incubated with IGF-1 or wortmannin for 30 min. While the antibody did not detect any phosphorylation signal in the 573 alanine mutant, it detected IGF-1 stimulated phosphorylation of serine 573 in wild type IRS-2. Again, wortmannin blocked the IGF-1-mediated phosphorylation of serine 573 (Fig. 5C). The antibody recognized different bands, which all corresponded to the IRS-2 protein according to the reblot. This recognition of different sub-species of the IRS-2 protein by immunoblotting is well in line with recent data [5]. To test whether the antibody recognized the homologue residue in human IRS-2 (serine 577) or in IRS-1 (serine 522 in rat and mouse numbering), HEK293 cells were co-transfected transiently with plasmids containing the insulin receptor and either mouse IRS-2, human IRS-2 or rat IRS-1 (Fig. 5D). The antibody detected phosphorylation of mouse IRS-2 Ser-573 and human IRS-2 Ser-577, but not IRS-1 Ser-522.

### Akt/PKB Activity is Important for Phosphorylation of Serine 573 and the Interaction of 14-3-3 with IRS-2

**Figure 6:**
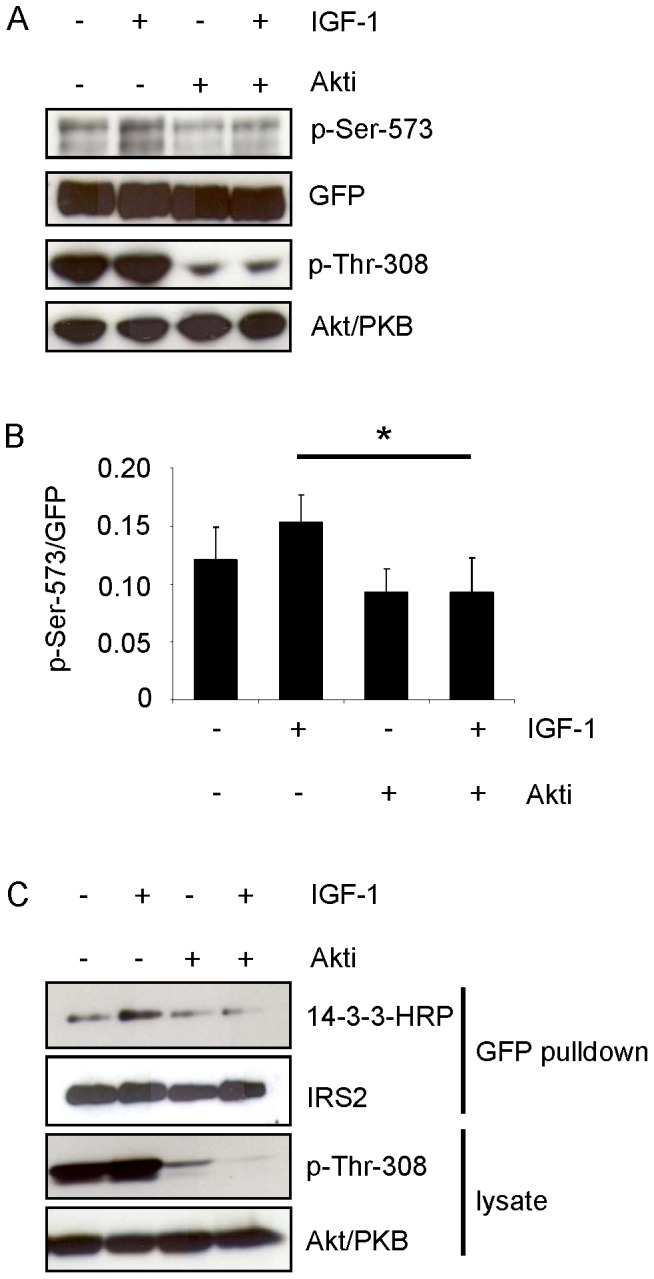
Inhibition of Akt/PKB reduces IGF-1-induced phosphorylation of serine 573 and 14-3-3 binding. *A*. Flp-In HEK293 cells stably expressing GFP-IRS2 were starved for serum and incubated for 30 min with 50 ng/ml IGF-1 or 1 µM Akti-1/2 alone, or Akti-1/2 preincubation followed IGF-1 stimulation. 100 µg of total protein was separated on 7.5% SDS gels and membranes were probed with p-Ser-573 and p-Thr-308 of Akt/PKB. Membranes were stripped and reprobed with respective antibodies for detection of protein levels. *B*. Effect of Akt/PKB inhibition on serine 573 phosphorylation was assessed by scanning densitometry of blots and normalization for protein (mean ± SEM; n = 3; *p<0.05 IGF-1 vs. Akti/IGF-1). *C*. GFP pulldown from Flp-In HEK293 cells stably expressing GFP-IRS2, stimulated as in *A*. 200 µg protein was pulled down and overlay assay followed reprobing with IRS-2 antibody. 100 µg of total protein was separated on 7.5% SDS gel and membrane was checked for p-Thr-308 phosphorylation and reprobed with Akt/PKB protein antibody.

The sequence surrounding serine 573 (RKRTYpS^573^) conformed to a potential consensus site for phosphorylation by Akt/PKB (RXRXXpS/T, X denotes any amino acid). To test whether Akt/PKB was involved in phosphorylation of serine 573, Flp-In HEK293 cells stably expressing GFP-IRS2 were incubated with an Akt/PKB inhibitor (Akti-1/2) before IGF-1 stimulation. The compound Akti-1/2 reduced the phosphorylation of Thr-308 of Akt/PKB and subsequently the IGF-1-stimulated phosphorylation of serine 573 (Fig. 6A, B). Of note, the Flp-In HEK293 cells showed phosphorylation of Thr-308-Akt/PKB in the serum starved condition, which may influence phosphorylation of serine 573. To further test whether Akt/PKB inhibition also influences 14-3-3 binding to IRS-2, we performed a GFP pulldown and subsequent overlay assay to visualize 14-3-3 binding to IRS-2. IGF-1 stimulation resulted in increased binding of 14-3-3 to IRS-2, which was prevented by incubation with Akti-1/2 before IGF-1 stimulation (Fig. 6C). These findings led us to the conclusion that Akt/PKB activity was necessary for serine 573 phosphorylation and the IGF-1-stimulated 14-3-3 binding to IRS-2.

### Monitoring 14-3-3 Interaction with IRS-2 and Serine 573 Phosphorylation in Rat Hepatoma Cells

**Figure 7:**
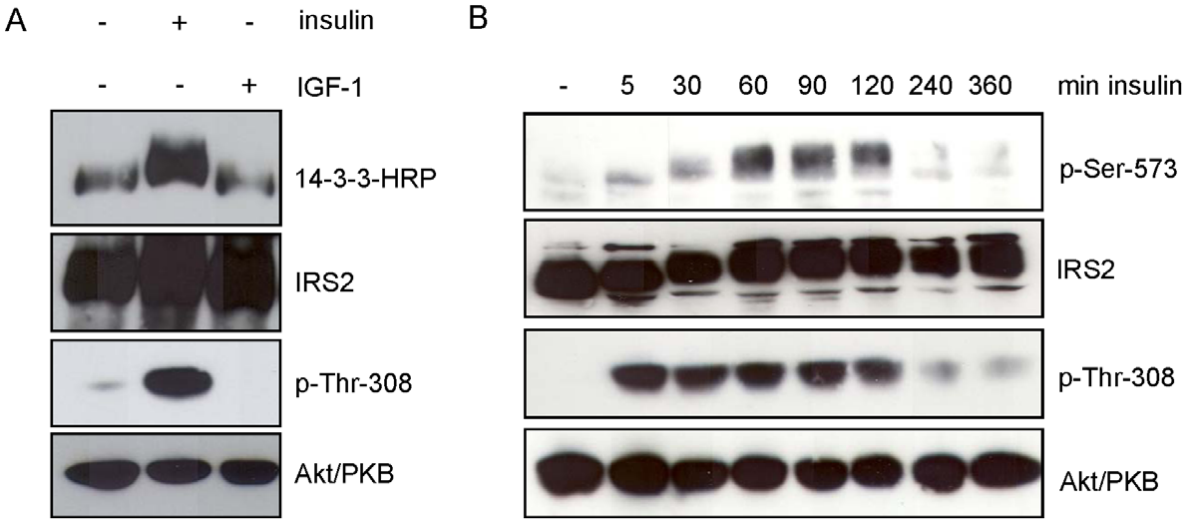
Insulin induces binding of 14-3-3 to endogenous IRS-2 and phosphorylates Ser-573 on IRS-2. *A.* Fao cells were starved for serum overnight and incubated for 30 min with either 10 nM insulin or 50 ng/ml IGF-1. 250 µg protein was immunoprecipitated with IRS-2 antibody and separated on a 5–15% gradient gel. Overlay assay followed stripping and reprobing with IRS-2 antibody as loading control. Successful stimulation is shown as phosphorylation of p-Thr-308 and corresponding Akt/PKB reblot. *B.* Fao cells were starved for serum overnight and stimulated with 10 nM insulin for the indicated time points. 100 µg of protein was separated on a 7.5% gel and membranes were probed with specific antibodies against p-Ser-573 of IRS-2 and p-Thr-308 of Akt/PKB. For loading control membranes were stripped and reprobed with protein antibody.

The preceding experiments were performed in transiently or stably transfected HEK293 cells. To test whether endogenously expressed IRS-2 was phosphorylated at serine 573 and whether 14-3-3 bound to IRS-2 upon IGF-1 or insulin stimulation, Fao (rat hepatoma) cells were treated with IGF-1 or insulin for 30 min. Insulin stimulation led to a strong interaction of 14-3-3 with IRS-2 and increased Akt/PKB phosphorylation at Thr-308 (Fig. 7A). Stimulation with IGF-1 did not result in activation of the PI 3-kinase-Akt/PKB pathway similar to previous data [5], probably due to low IGF-1 receptor expression levels and did not lead to increased 14-3-3 binding above basal levels. Therefore, insulin stimulation was used to monitor phosphorylation of serine 573 in a time course experiment in Fao cells. Fao cells were starved for serum and incubated with insulin for the indicated time points. Serine 573 showed a slight basal phosphorylation, which modestly increased after 5 and 30 min and peaked at 60, 90 and 120 min of insulin stimulation before decreasing (Fig. 7B) again.

### Positive Influence of S573A Mutant on Akt/PKB Phosphorylation

**Figure 8:**
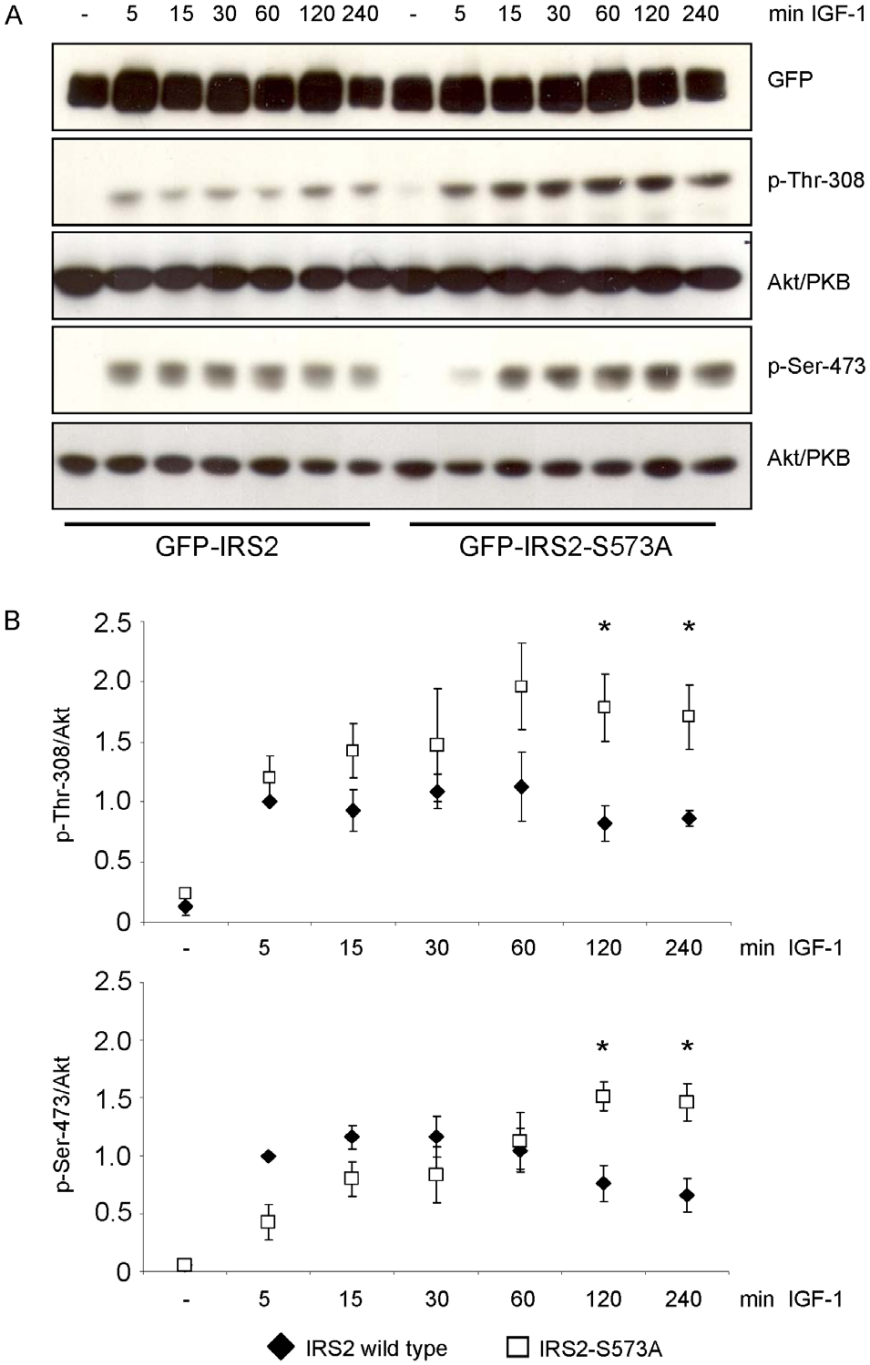
Ser-573 influences phosphorylation of Akt/PKB. *A*. HEK293 cells transiently expressing GFP-IRS2 or GFP-IRS2-S573A were stimulated with 50 ng/ml IGF-1 for the indicated time points. 40 µg of total protein was separated on 7.5% SDS gels and membranes were incubated with GFP antibody to ensure equal expression levels and with p-Thr-308 and p-Ser-473 antibody respectively. Corresponding Akt/PKB reblots are shown. *B*. Densitometric analyses of Akt/PKB phosphorylation. Black diamonds represent IRS-2 wild type, white squares IRS2-S573A mutant. Phosphorylation was normalized against total protein and IRS-2 wild type stimulated with IGF-1 for 5 min was set as 1 (mean ± SEM; n = 3; *p<0.05 IRS-2 120 min vs. S573A 120 min; IRS-2 240 min vs. S573A 240 min).

To investigate if the phosphorylation of serine 573 had an influence on downstream signaling of the IGF-1/insulin signaling cascade, the phosphorylation of Thr-308 and Ser-473 of Akt/PKB in HEK293 cells transfected transiently with GFP-IRS2 or GFP-IRS2-S573A was assessed after incubation with IGF-1 for 5, 15, 30, 60, 120 and 240 min. IRS-2 wild type expressing cells showed induction of phosphorylation of Thr-308 and Ser-473 after 5 min of IGF-1 stimulation and this stimulation was decreased after 120 min (Fig. 8). In contrast, this downregulation was impaired in the S573A mutant expressing cells. This data suggested that phosphorylation of serine 573 negatively regulated IGF-1 signaling.

### Involvement of Other IGF-1/insulin-dependent 14-3-3 Binding Sites on IRS-2

**Figure 9:**
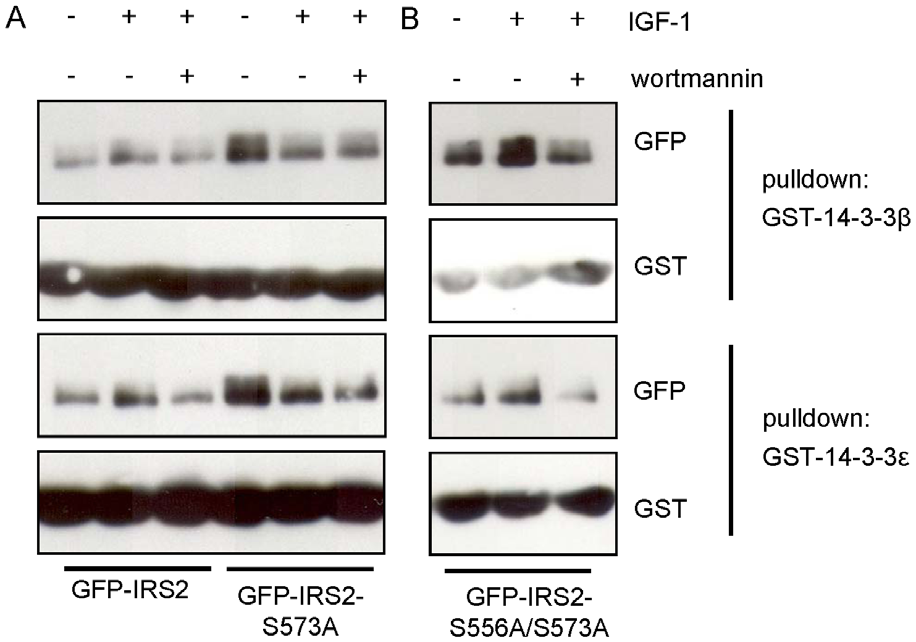
GST pulldown of transiently transfected IRS-2 and mutants. *A* and *B*. GFP-IRS2, GFP-IRS2-S573A and GFP-IRS2-S556A/S573A were expressed transiently in HEK293 cells and cells were stimulated with 50 ng/ml IGF-1 for 30 min or after preincubation with 100 nM wortmannin for 30 min. Lysates were incubated with 2 µg GST-14-3-3β or ε for 2 hours. Samples were separated on 7.5% SDS gels and membranes were probed with GFP and GST antibodies.

The results of the overlay assay using alanine mutants of the phosphorylation sites between amino acids 301–600, identified in the mass spectrometric approach, did not indicate a second site relevant for the 14-3-3 binding to IRS-2 (Fig. 4). However, with a differential 14-3-3 affinity approach using insulin-stimulated HeLa cells and the yeast 14-3-3 isoforms BMH1 and BMH2 as capture proteins, Dubois et al. identified IRS-2 as a 14-3-3 interaction protein and by mass spectrometry also identified an IRS-2 derived peptide containing a phosphorylated serine corresponding to serine 556 [36]. To study the potential involvement of this site together with serine 573 in 14-3-3 binding we applied a GST pulldown assay, where cell lysates were mixed with GST tagged 14-3-3β or 14-3-3ε. Western blot membranes were incubated with GST antibody to ensure equal pulldown and with GFP to visualize the amount of protein that interacted with GST-14-3-3. First, we applied the GST pulldown for the single serine 573 to alanine mutant of IRS-2 to confirm serine 573 as 14-3-3 binding site. Unexpectedly, the GST pulldown experiments showed no reduced interaction of IRS2-S573A with 14-3-3 (Fig. 9A) in contrast to the results of the overlay assays in Figure 4. The interaction was also not reduced using the single S556A mutant (data not shown) and also a double GFP-IRS2 mutant S556A/S573A failed to disrupt 14-3-3 binding (Fig. 9B). Together, these results may either reflect restrictions of the GST pulldown in the context with our protein constructs of IRS-2 and 14-3-3 or give a hint for an additional binding site of 14-3-3 in IRS-2 that does not influence the binding detectable in the overlay assay but the binding visualized in the GST pulldown.

## Discussion

IRS-1 and -2 as intracellular signaling nodes contain numerous serine and threonine residues that can be modified by protein kinases and protein phosphatases. For IRS-1 this has been shown extensively [8], [11], [38]–[40]. Additionally, the interaction of IRS-1 and 14-3-3 has been investigated in some studies. 14-3-3 binding to IRS-1 was found to be influenced by insulin in HepG2 cells [37] but not in 3T3-L1 adipocytes [41]. Xiang et al. proposed a role for 14-3-3 in IRS-1 trafficking in COS-7 cells [42], whereas Oriente et al. and Kosaki et al. observed an influence of 14-3-3 on IRS-1 associated kinases and their activity in NIH-3T3 fibroblasts and 3T3-L1 adipocytes [41], [43].

Less is known about individual serine/threonine phosphorylation sites on IRS-2, the interaction of 14-3-3 with IRS-2 and the influence on cellular metabolism. The IRS-2 interaction with 14-3-3 proteins has been documented in large-scale proteomic studies, but only as one of many interaction partners without any further characterization [44]–[46]. Ogihara et al. showed interaction of overexpressed IRS-2 and 14-3-3 in Sf9 cells [37] and Dubois et al. showed IRS-2 as 14-3-3 interaction partner upon IGF-1 stimulation involving the PI 3-kinase pathway in HEK293 cells [36].

This interaction was verified here in different cell culture models and in the liver of mice *in vivo*. In addition, we present here a pattern of 24 phospho-sites of IRS-2 after IGF-1 stimulation and the IGF-1/insulin-regulated 14-3-3 binding region on IRS-2 involving the novel IRS-2 serine phosphorylation site 573. We showed that upon insulin or IGF-1 stimulation 14-3-3 associated with IRS-2 in transiently transfected HEK293 cells, in Flp-In HEK293 cells that stably expressed IRS-2 and in Fao cells, which expressed IRS-2 endogenously. In addition, we could provide evidence that the IGF-1/insulin-dependent activation of the PI 3-kinase-Akt/PKB pathway was necessary for the IGF-1/insulin-stimulated phosphorylation of serine 573 and the 14-3-3 binding of IRS-2. Our data suggest that serine 573 potentially is involved in the negative regulation of IGF-1/insulin signaling, since the 573 alanine mutant led to increased Akt/PKB signaling. In mice liver 14-3-3 associated with IRS-2 upon insulin stimulation and refeeding, thus indicating the physiological occurrence of this interaction.

We used the IRS-2 mouse sequence to identify novel phosphorylation sites on IRS-2 and the mass spectrometry approach revealed 24 phosphorylated serine/threonine residues. These sites seem to be conserved in human IRS-2 since all sites could also be found in the human IRS-2 protein sequence. In addition, certain sites could be found in the corresponding human and mouse protein sequences of IRS-1. Phosphorylation of serine 675 and serine 907 after insulin stimulation has already been investigated and described by our group [5]. Serine 303 has a homologue in the human IRS-2 protein sequence - serine 270. This site has been described as substrate for S6K1 and as part of TNF-α-induced insulin resistance [47]. Little is known about the other sites that were identified by our screen. Truncated versions of the IRS-2 protein, spanning 5 different segments, revealed the area residing between amino acid position 301 and 600 to be important for 14-3-3 binding. Within this region our mass spectrometric approach identified several phosphorylated residues, but only mutation of serine 573 to alanine abrogated the 14-3-3 interaction with IRS-2 in the overlay assay. Ser-573 and the surrounding amino acids (RKRTYS^573^SLTT) do not completely conform to a 14-3-3 binding motif with the lack of proline at position +2 relative to the phosphorylated serine being the most prominent feature to be different. However, a proline in positin +2 is found in only 50% of the target sites as has been reported by Johnson et al [48]. In addition, if the target sequence would match the consensus motif completely, the strength of binding would not give appropriate regulation of this protein [49]. The phosphorylation of serine 573 was increased upon IGF-1/insulin stimulation in a PI 3-kinase-Akt/PKB-dependent manner, thus under the same conditions when the 14-3-3/IRS-2 interaction took place. Notably, pharmacological inhibition of PI 3-kinase using wortmannin or PI-103 or inhibition of Akt/PKB using Akti-1/2 prevented the enhanced phosphorylation of serine 573 and the increased 14-3-3 binding of IRS-2.

We focused on the characterization of serine 573 since a physiological importance of serine 573 is suggested by the fact that sequence alignments of different mammalian species showed complete sequence accordance, and even an amphibian sequence showed partial accordance. We could observe phosphorylation of serine 573 of mouse IRS-2 with insulin and IGF-1 and also phosphorylation of the corresponding residue serine 577 in human IRS-2. A time course of serine 573 phosphorylation in Fao cells showed peak phosphorylation levels after 60, 90 and 120 min of insulin treatment. Moreover, mutation of this site to alanine displayed clear effects on IGF-1/insulin signal transduction.

IRS-1 and IRS-2 display a similar architecture and partially overlapping functions, therefore the IRS-1 sequence was screened for a homologue position corresponding to serine 573 of IRS-2. Giraud et al. described the homologue serine 522 in IRS-1 [50]. They showed insulin-dependent phosphorylation of serine 522 in L6 myoblasts and myotubes and identified Akt/PKB or a kinase downstream of Akt/PKB as kinase for its phosphorylation and provided evidence for a negative role of serine 522 phosphorylation. Serine 573 on IRS-2 also resides in an Akt/PKB consensus motif (RXRXXpS/T). Treating cells with an Akt/PKB inhibitor resulted in significantly reduced phosphorylation of serine 573 and further experiments showed increased Akt/PKB phosphorylation upon IGF-1 stimulation in the S573A mutant compared to IRS-2 wild type. In conclusion, our data suggested comparable regulation and function of the homologue serine 573 and 522 in IRS-2 and IRS-1, but the implication of serine 522 in 14-3-3 binding of IRS-1 has not been reported yet.

The data of the overlay assay indicated that only one phosphorylated residue on IRS-2 was sufficient to trigger IGF-1/insulin-dependent 14-3-3 interaction with IRS-2. This would be in line with several other studies which also identified single phosphorylated residues as 14-3-3 interaction site [26]–[28]. However, there are also other reports that showed 14-3-3 binding to two simultaneously phosphorylated serine/threonine residues on the same target protein (see [48] for a collection of reported binding partners). Serine 556 appeared in the study from Dubois et al. to be phosphorylated after insulin stimulation in HeLa cells and we identified the same residue in our mass spectrometric approach. This opened the possibility that perhaps serine 556 and serine 573 were 14-3-3 binding sites. The S556A mutant showed regulated binding of 14-3-3 in the overlay assay, hence arguing against S556A as a 14-3-3 binding site. To support these findings with another methodical approach GST pulldown experiments were conducted. Unexpectedly, both single mutants S556A and S573A did interact with GST-14-3-3 and also the double mutant S556A/S573A failed to disrupt the interaction. The cause of this discrepancy between the overlay assay and GST pulldown cannot be explained yet, but we speculate that the S573A mutant probably failed to disrupt interaction of 14-3-3 with IRS-2 in the GST pulldown due to the presence of other proteins stabilizing 14-3-3 binding or preventing regulated binding of exogenously added GST-14-3-3 protein. Of course a second IGF-1/insulin-dependent 14-3-3 binding site on IRS-2 cannot be excluded.

How can the persuasive result of the overlay assay be explained? A possibility is partial renaturation of the IRS-2 protein on the nitrocellulose membrane. The renaturation process probably was effective enough to enable binding of 14-3-3 to the phospho-serine 573 motif, whereas the second binding site might not have been easily accessible and full renaturation of the IRS-2 protein would have been required. This may have led to abrogation of 14-3-3 binding in the S573A mutant in the overlay assay, despite the presence of a second binding site.

The 14-3-3 protein family shows high overall sequence conservation, in the central binding groove the residues are strictly conserved [20] and the phosphopeptide bonds are build with 3 absolutely conserved residues [51]. In addition, the high level of conservation is emphasized by the fact that 14-3-3 isoforms from yeast, plant and mammals are functionally interchangeable [18]. The aforementioned properties show that the two 14-3-3 isoforms β and ε used in this study for the GST pulldowns and 14-3-3ζ for the overlay assays were adequate to investigate 14-3-3 binding sites on IRS-2.

How could the interaction of IRS-2 with 14-3-3 proteins influence downstream insulin signaling? The rigid structure of the 14-3-3 proteins led to the hypothesis that 14-3-3 proteins provide themselves as an anvil whereon the target protein can be restructured/reshaped [18]. Experimental data from Datta et al. showed that upon 14-3-3 binding to serine 316 of BAD (Bcl-2-associated death promoter) the accessibility of serine 155 of BAD for other kinases increased [30]. GlobPlot® analysis revealed unstructured regions in the IRS-2 protein, which could potentially be restructured/reshaped upon 14-3-3 binding. Since IRS-2 functions as a multi-adapter protein, thereby linking the insulin receptor and other receptor tyrosine kinases with intracellular effector molecules, 14-3-3 proteins could modulate the interactions with binding partners by binding to IRS-2 upon phosphorylation of serine 573 and a possible yet to be discovered second binding site.

In summary, we demonstrate the identification of 24 phosphorylation sites including novel sites on IRS-2 and mapped the IGF-1/insulin-dependent 14-3-3 binding region. Serine 573 as novel phosphorylation site was shown to be part of IGF-1/insulin-dependent interaction between 14-3-3 and IRS-2 and its phosphorylation may have an inhibitory role in IGF-1/insulin signaling. The evidence of the interaction between 14-3-3 proteins and the signal transducer IRS-2 *in vivo* opens several novel perspectives in the (patho)physiological regulation of the biological function of IRS-2 including its role in metabolic disorders such as insulin resistance and type 2 diabetes.

## Materials and Methods

### Ethics Statement

The animal experiments were conducted in accordance with the national guidelines of laboratory animal care and were approved by the local governmental commission for animal research (M6/08, Regierungspraesidium Tuebingen, Baden-Wuerttemberg, Germany).

### Materials

Flp-In HEK293 cells were from Invitrogen (Karlsruhe, Germany), Fao and HEK293 cells from The European Collection of Cell Cultures (Salisbury, UK). Insulin, IGF-1 and phosphatase inhibitors (sodium fluoride, sodium pyrophosphate, sodium orthovanadate and β-glycerophosphate) were obtained from Sigma (Munich, Germany), wortmannin and Akt/PKB inhibitor (Akti-1/2) from Calbiochem (Schwalbach, Germany). IRS-2 polyclonal protein antibody (06–506) was from Millipore (Schwalbach, Germany), Akt/PKB protein antibody (610861) from BD Transduction Laboratories (Erembodegem, Belgium), GFP (green fluorescent protein) polyclonal protein antibody (8334), 14-3-3 (C-17) (732) and 14-3-3 K-19 (629) from Santa Cruz (Santa Cruz, USA) and phosphospecific Thr-308-Akt/PKB (9275) as well as Ser-473-Akt/PKB (9271) from Cell Signaling (Boston, USA). Recombinant human 14-3-3ζ-HRP was from R&D Systems (Minneapolis, USA). Trypsin gold, mass spectrometry grade was from Promega (Madison, USA), 4–12% Bis-Tris mini gels and Colloidal Blue Staining Kit were from Invitrogen (Karlsruhe, Germany). Protease Inhibitor Cocktail Tablets were from Roche and the Protein Assay was from BioRad (Munich, Germany). Protein A Sepharose, Sepharose 4B and GST (glutathione S-transferase) antibody (27-4577-01) were from GE Healthcare Europe (Munich, Germany). GFP-Trap® was obtained from Chromotek (Martinsried, Germany).

### Cell Culture and Transfection

All cell lines were kept in a 5% CO_2_-atmosphere with 95% humidity at 37°C. HEK293 and Flp-In HEK293 cells were cultivated in DMEM with 4.5 g/l glucose, supplemented with 10% FBS, 1% glutamine, 100 U/ml penicillin and 100 U/ml streptomycin. Fao cells were cultivated in RPMI 1640 supplemented with 10% FBS, 1% glutamine, 100 U/ml penicillin and 100 U/ml streptomycin. Flp-In HEK293 and HEK293 cells were transfected either transiently with polyethylenimine or stably with the Calcium Phosphate method [52] and positive clones were selected with Hygromycin B. Fao cells were used at confluency. Stimulation of cells was carried out after starvation for serum with various substances as described in detail in the results part/legends.

### Cell Lysis, Immunoprecipitation and Western Blotting

Cells were lysed with lysis buffer consisting of 50 mM HEPES, 150 mM NaCl, 1.5 mM MgCl_2_, 1 mM EDTA, 10% glycerol, 1% Triton-X-100, set to pH 7.5, containing phosphatase inhibitors (10 mM NaF, 5 mM sodium pyrophosphate, 10 mM sodium orthovanadate, 10 mM β-glycerophosphate). Lysates were cleared by centrifugation for 5 min at 4°C with 16000 *g*. IRS-2 was immunoprecipitated with 2 µg IRS-2 antibody coupled to Protein A Sepharose for 4 hours at 4°C. 14-3-3 was immunoprecipitated by mixing lysates with 2 µg 14-3-3 antibody for 3 hours and adding 30 µl Protein G Sepharose for another hour. The Sepharose pellet was washed two times with lysis buffer and resuspended in 5x Laemmli (1 M Tris-HCl, pH 6.8, 50% glycerin, 10% SDS, 5.5% β-mercaptoethanol). Proteins were separated on either 7.5% or 5–15% SDS gels followed by transfer onto nitrocellulose membranes using semi-dry Western blot. Western blotting and enhanced chemiluminescence was described previously [53].

### Plasmid Constructs

All plasmids to express GFP tagged proteins were generated at the University of Dundee (UK). All PCR and mutagenesis reactions were carried out using KOD Hot Start DNA polymerase (Novagen). The coding region for mouse IRS-2 (NM_001081212.1) was amplified from a pRK5 IRS-2 vector (kindly provided by M.F. White, Boston, MA, USA) using primers 5′-gaggatccatggctagcgcgcccctgcctg-3′ and 5′-ctgcggccgctcgagtcactctttcacgactgtggcttccttc-3′, cloned into vector pSC-B (Stratagene) and sequenced. IRS-2 was sub-cloned from this plasmid into pcDNA5/FRT/TO-GFP as a BamH1/Not1 insert to give vector pcDNA5/FRT/TO-GFP-IRS2 in which IRS-2 is tagged with GFP at the N-terminus. For truncated IRS-2 versions containing the IRS-2 sequence from amino acid 1–300 and 1–600 a stop codon was created after position 300 (sense: 5′-gagttccggcctcgctgaaagagtcagtcgtcc-3′; antisense: 5′-ggacgactgactctttcagcgaggccggaactc-3′) or 600 (sense: 5′-ctcatgagggccacctgatctggtagttcaggtc-3′; antisense: 5′-gacctgaactaccagatcaggtggccctcatgag-3′) respectively. For the constructs 301–1321 and 601–1321 a BamHI restriction site was created prior positions 301 and 601 and the obtained inserts were ligated into pcDNA5/FRT/TO-GFP. Construct 301–600 was generated by creating a stop codon after position 600 and pcDNA5/FRT/TO-GFP-IRS2-301–1321 served as template. pcDNA5/FRT/TO-GFP-IRS2 served as template for the point mutation of serine 573 to alanine (sense: 5′-gaggaagaggacttatgccctaaccacgcctg-3′; antisense: 5′-caggcgtggttagggcataagtcctcttcctc-3′) and for the point mutation of serine 556 to alanine (sense: 5′-ccttaccgtagggtcgcaggggatggggccc-3′; antisense: 5′-gggccccatcccctgcgaccctacggtaagg-3′). pcDNA5/FRT/TO-GFP-IRS2-S556A served as template for the generation of double mutant S556A/S573A (sense: 5′- gaagaggacttatgccctaaccacgcctg-3′; antisense: 5′-caggcgtggttagggcataagtcctcttc-3′). DNA sequencing was performed by The Sequencing Service, College of Life Sciences, University of Dundee (www.dnaseq.co.uk). The plasmid construct for human IRS-2 was from Calum Sutherland (University of Dundee).

### GFP Pull Down and Far Western Blot

All centrifugation steps were carried out at 4°C for 2000 *g* for 1 min. Precleared lysates were transferred into fresh tubes containing a 50% slurry of a mixture of Protein A Sepharose and GFP-Trap® and samples were mixed for 2 hours at 4°C. After washing twice with high salt buffer (50 mM Tris, pH 7.5, 150 mM NaCl) and twice with no salt buffer (50 mM Tris, pH 7.5, 1 mM EGTA, 0.1% mercaptoethanol), the pellet was resuspended in 5x Laemmli buffer. Proteins were separated on 5–15% gradient gels and transferred onto nitrocellulose membranes. For Far Western blot membranes were blocked in 5% milk powder in TBS-T (25 mM Tris, pH 7.4, 0.15 M NaCl, 0.1% Tween 20) for 1 hour at room temperature. Incubation of the membrane overnight at 4°C with recombinant human 14-3-3ζ-HRP was followed by washing with TBS-T and visualization using ECL. Incubation of membranes with 14-3-3ζ-HRP will be referred to as overlay assay.

### Expression of GST Fusion Proteins and GST Pulldown

GST-14-3-3β and GST-14-3-3ε constructs were a kind gift from Angelika Hausser (University of Stuttgart). The GST-14-3-3 plasmids were propagated in *E. coli* and protein expression was induced by 1 mM IPTG (isopropyl β-D-1-thiogalactopyranoside) for 5 hours. Bacterial cells were harvested at 4°C by centrifugation with 4500 *g* for 15 min and disrupted by applying 40 strokes with a probe-type sonicator. After adding Triton-X-100 to a final concentration of 1% the suspension was centrifuged at 4500 *g* for 20 min at 4°C. GST-14-3-3 proteins were coupled to 50% slurry of glutathione Sepharose 4B. The amount of GST-14-3-3 was determined by SDS-PAGE followed by Coomassie staining. Lysates were incubated with 2 µg GST-14-3-3 for 2 hours in lysis buffer at 4°C. Beads were washed three times with lysis buffer, resuspended in 5x Laemmli and denaturated at 95°C for 5 min.

### Polyclonal Antibody Against Phosphorylated Serine 573

The peptide CLRKRTYS*LTTPAR (residues 567–579, *indicates phosphorylated serine) was used to generate a phosphospecific antibody against phosphorylated residue 573 of mouse IRS-2. The phosphopeptide was conjugated to KLH (keyhole limpet haemocyanin) and BSA using MBS (*m*-maleimidobenzoic acid *N*-hydroxysuccinimide ester) according to the method of Harlow and Lane [54]. Five aliquots containing 0.5 mg of each peptide conjugate were sent to the Scottish Blood Transfusion Service, Penicuik, Scotland. Injection into sheep at monthly intervals was followed by collecting serum at monthly intervals and purification by affinity chromatography against the appropriate phosphorylated peptide.

### Sample Preparation for Mass Spectrometry

HEK293 cells were seeded onto 10 cm diameter plates, transfected with 10 µg DNA of GFP or GFP-IRS2 and 30 µg polyethylenimine in 25 mM HEPES/plate, starved for serum and stimulated with 50 ng/ml IGF-1 for 30 min and after preincubation with 1 µM PI-103. Cell lysis was done in lysis buffer (50 mM Tris-HCl, pH 7.5, 1 mM EGTA, 1 mM EDTA, 1% Triton-X-100, 1 mM sodium orthovanadate, 50 mM sodium fluoride, 5 mM sodium pyrophosphate) containing a Protease Inhibitor Cocktail tablet. Protein determination was followed by immunoprecipitation of GFP proteins from 10 mg of total protein using 15 µl GFP Trap® and separation on precast 4–12% Bis-Tris gels. Staining overnight using the Colloidal Blue Staining Kit followed cutting of IRS-2 bands and transfer into 1.5 ml reaction tubes. Gel pieces were washed sequentially with ultrapure H_2_O, 50% methanol/H_2_O, 0.1 M NH_4_HCO_3_ and methanol/50 mM NH_4_HCO_3_. Proteins were incubated with 10 mM DTT/0.1 M NH_4_HCO_3_ at 65°C for 45 min, followed by alkylation with 50 mM iodacetamide/0.1 M NH_4_CO_3_ in the dark for 20 min at room temperature. Washing of gel pieces using 50 mM NH_4_HCO_3_ and 50 mM NH_4_HCO_3_/50% methanol was followed by shrinking the pieces with methanol for 15 min and drying in a Speed-Vac. Gel pieces were soaked in 20 mM triethylammonium bicarbonate containing 5 µg/ml trypsin and shaken at 30°C overnight. Methanol was added and samples shaken for 15 min before the supernatant was removed and samples were dried in a Speed-Vac. Residual peptides were extracted from the gel pieces with 50% methanol/2.5% formic acid and the supernatant was combined with the first dried extract and dried completely.

### Mass Spectrometry

IRS-2 tryptic digests were analysed by LC-MS (liquid chromatography mass spectrometry) on a LTQ-orbitrap classic mass spectrometer system (Thermo Fisher Scientific, Schwerte, Germany) coupled to a Proxeon Easy-LC HPLC system. The peptide mixtures were loaded onto a nanoseparations C_18_ guard column (0.1×20 mm) equilibrated in 0.1% formic acid/water at 5 µl/min and then separated on a 0.075×150 mm PepMap C_18_ column, equilibrated in 0.1% formic acid/water (LC Packings, Amsterdam, Netherlands). Peptides were eluted with a 100 min discontinuous gradient of acetonitrile/0.1% formic acid at a flow rate of 300 nl/min. The column outlet was connected to a Thermo Fisher Nanospray 1 source fitted with a New Objective FS360 20-10 uncoated emitter and a voltage of 1.3 kV was applied to the emitter. The orbitrap was set to analyse the survey scans (m/z 350–2000) at 60.000 resolution and top 5 ions in each duty cycle (minimum ion intensity of 50000 cps), were selected for MSMS in the LTQ linear ion trap with multistage activation. Ions were excluded for 30 s after 2 occurrences. The raw files were converted into mascot generic files using Raw2msm (a gift from M. Mann, Martinsried, Germany). The MSMS spectra (mgf file) were searched against SwissProt database using the Mascot search engine (Matrix Science) run on an in-house server using the following criteria: peptide tolerance = 10 ppm; trypsin as the enzyme; carboxyamidomethylation of cysteine as a fixed modification with oxidation of methionine and phosphorylation of serine, threonine and tyrosine as a variable modification. Any MSMS spectra that could be assigned to a phosphopeptide was inspected manually using QualBrowser software. IRS-2 tryptic digests were also analysed by LC-MS with precursor of 79 scanning on a 4000 Q-Trap system (Applied Biosystems, Carlsbad, US) as described previously [55]. Extracted ion chromatograms for all detected phosphopeptides were generated using Analyst 1.4.1 software.

### Animal Studies

Male C57Bl/6 mice were from Charles River (Sulzfeld, Germany), maintained at a 12 hours dark/light cycle under standard chow. Liver was homogenized at 4°C in lysis buffer (50 mM Tris-HCl, pH 7.5, 150 mM NaCl, 1% Triton-X-100, containing Protease Inhibitor Complete Tablet and phosphatase inhibitors). Homogenates were cleared after 30 min of solubilization on ice by 3 centrifugation steps at 16 000 *g* for 10 min at 4°C. All procedures were approved by the local Animal Care and Use committee.

### Statistical Analyses

Data are presented as means ± SEM. Unpaired student’s t-test was employed and a result was considered significant if p<0.05.

## Supporting Information

Table S1 **Phosphopeptides detected by mass spectrometry that are common to IRS-2 isolated from serum starved and IGF1 treated cells.** GFP-IRS2 was expressed transiently in HEK293 cells and cells were either left unstimulated or stimulated with IGF-1 for 30 minutes. Using GFP-Trap® IRS-2 was purified from total cell lysate and after SDS-PAGE and Coomassie staining, the bands corresponding to IRS-2 were cut and peptides were digested using trypsin (M = molecular mass of the peptide in Dalton, m/z = mass to charge). A mass accuracy of 10 ppm was used in database searches and phosphosites were identified by MS/MS analysis and manual inspection of the spectra. The sequence coverage was 92%. The phosphorylated residues are indicated with pS or pT.
(DOC)

Table S2 **Phosphopeptides detected by mass spectrometry that are unique to IRS-2 isolated from IGF-1-treated cells.** Samples were prepared as in Table S1. The phosphorylated residues are indicated with pS or pT.
(DOC)
