# Supplementary material for: Identification of the Amino Acids 300–600 of IRS-2 as 14-3-3 Binding Region with the Importance of IGF-1/Insulin-Regulated Phosphorylation of Ser-573
Source: PLoS One. 2012 Aug 17;7(8):e43296. doi: 10.1371/journal.pone.0043296 (PMC3422239; doi:10.1371/journal.pone.0043296)
Supplement: Table S1 — Phosphopeptides detected by mass spectrometry that are common to IRS-2 isolated from serum starved and IGF1 treated cells. GFP-IRS2 was expressed transiently in HEK293 cells and cells were either left unstimulated or stimulated with IGF-1 for 30 minutes. Using GFP-Trap® IRS-2 was purified from total cell lysate and after SDS-PAGE and Coomassie staining, the bands corresponding to IRS-2 were cut and peptides were digested using trypsin (M = molecular mass of the peptide in Dalton, m/z = mass to charge). A mass accuracy of 10 ppm was used in database searches and phosphosites were identified by MS/MS analysis and manual inspection of the spectra. The sequence coverage was 92%. The phosphorylated residues are indicated with pS or pT. (DOC) [file pone.0043296.s001.doc]

Supporting Table S1

*Phosphopeptides detected by mass spectrometry that are common to IRS-2 isolated from serum starved and IGF-1- treated cells.*

| **Residue** | **m/z** | **M** | **Sequence (ion score > 20)** |
| --- | --- | --- | --- |
| 66 | 971,9192 | 1941,8218 | GPGTGGDEASAAGG**pS**PPQPPR (105) |
| 303 | 1010,9786 | 2019,9375 | SK**pS**QSSGSSATHPISVPGAR (107) |
| 347 | 611,7894 | 1221,5642 | TDSLAA**pT**PPAAK (50) |
| 385/388 | 1038,1247 | 3111,3482 | TASEGDGGAAGGAGTAGGRPMSVAG**pS**PL**pS**PGPVR (98) |
| 388 | 1011,4705 | 3031,3819 | TASEGDGGAAGGAGTAGGRPMSVAGSPL**pS**PGPVR (97) |
| 401 | 747,8305 | 1493,6446 | SH**pT**LSAGCGGRPSK (57) |
| 517 | 775,3495 | 1548,6820 | SN**pT**PESIAETPPAR (55) |
| 556 | 684,7994 | 1367,5830 | RV**pS**GDGAQDLDR (62) |
| 573 | 623,3026 | 1244,5914 | RTY**pS**LTTPAR (38) |
| 675 | 904,8615 | 1807,7045 | SDDYMPm**pS**PTSVSAPK (54) |
| 722 | 883,8343 | 1765,6502 | A**pS**SPAESSPEDSGYmR (73) |
| 727/728 | 915,8195 | 1829,6216 | ASSPAE**pSpS**PEDSGYMR (60) |
| 762 | 1522,1939 | 3041,3841 | LLPNGDYLNM**pS**PSEAGTAGTPPDFSAALR (82) |
| 907 | 903,3926 | 1804,7669 | **pS**PGEYINIDFGEAGTR (72) |
| 968 | 898,8778 | 1795,7375 | SPL**pS**DYmNLDFSSPK (67) |
| 977 | 930,8636 | 1859,7089 | SPL**pS**DYMNLDFSpSPK (75) |
| 999 | 1632,7888 | 4895,3365 | SGDTVGSMDGLL**pS**PEASSPYPPLPPRPSTSPSSLQQPLPPAPGDLYR (50) |
| 1089 | 470,2310 | 938,4474 | VA**pS**PTSGLK (61) |
| 1151 | 1308,5862 | 2615,1541 | HSSETFSSTTTVTPV**pS**PSFAHNSK (86) |
| 1165 | 696,8173 | 1391,6194 | HNSA**pS**VENVSLR (84) |
| 1190 | 1117,5376 | 3349,5827 | SSEGSSTLGGGDEPPT**pS**PGQAQPLVAVPPVPQAR (101) |

GFP-IRS2 was expressed transiently in HEK293 cells and cells were either left unstimulated or stimulated with IGF-1 for 30 minutes. Using GFP-Trap® IRS-2 was purified from total cell lysate and after SDS-PAGE and Coomassie staining, the bands corresponding to IRS-2 were cut and peptides were digested using trypsin (M = molecular mass of the peptide in Dalton, m/z = mass to charge). A mass accuracy of 10 ppm was used in database searches and phosphosites were identified by MS/MS analysis and manual inspection of the spectra. The sequence coverage was 92 %. The phosphorylated residues are indicated with pS or pT.
