# Supplementary material for: Identification of the Amino Acids 300–600 of IRS-2 as 14-3-3 Binding Region with the Importance of IGF-1/Insulin-Regulated Phosphorylation of Ser-573
Source: PLoS One. 2012 Aug 17;7(8):e43296. doi: 10.1371/journal.pone.0043296 (PMC3422239; doi:10.1371/journal.pone.0043296)
Supplement: Table S2 — Phosphopeptides detected by mass spectrometry that are unique to IRS-2 isolated from IGF-1-treated cells. Samples were prepared as in Table S1. The phosphorylated residues are indicated with pS or pT. (DOC) [file pone.0043296.s002.doc]

Supporting Table S2

*Phosphopeptides detected by mass spectrometry that are unique to IRS-2 isolated from IGF-1-treated cells.*

| **Residue** | **m/z** | **M** | **Sequence (ion score > 20)** |
| --- | --- | --- | --- |
| 305 | 903,4133 | 1804,8105 | SQ**pS**SGSSATHPISVPGAR (89) |
| 1266 | 779,6802 | 2336,0182 | GEQGSLAQSQPQPGDKN**pS**WSR (34) |

Samples were prepared as in Supporting Table S1. The phosphorylated residues are indicated with pS or pT.
